# Integrated Proteomics and Metabolomics Reveal Regulatory Pathways Underlying Quality Differences Between Wild and Cultivated *Ophiocordyceps sinensis*

**DOI:** 10.3390/jof11070469

**Published:** 2025-06-20

**Authors:** Chuyu Tang, Tao Wang, Yuejun Fan, Jie Wang, Mengjun Xiao, Min He, Xiyun Chang, Yuling Li, Xiuzhang Li

**Affiliations:** 1 State Key Laboratory of Plateau Ecology and Agriculture, Qinghai Academy of Animal and Veterinary Sciences, Qinghai University, Xining 810016, China; chuyutang0410@163.com (C.T.); 13085500761@163.com (T.W.); fanyuejun_79@163.com (Y.F.); wangjie08142023@163.com (J.W.); 15574237597@163.com (M.X.); himi1228@163.com (M.H.); 2 The Department of Pharmacy, Qinghai Institute of Health Sciences, Xining 810016, China; 15909715156@163.com

**Keywords:** cultivated, metabolomics, *Ophiocordyceps sinensis*, proteomics, quality, wild

## Abstract

*Ophiocordyceps sinensis*, is an entomopathogenic fungus renowned for its medicinal properties, thriving in the frigid and high-altitude regions of the Qinghai–Tibet plateau. Given the limited availability of wild resources and the increasing recognition of their medicinal value, the cultivation of *O. sinensis* was initiated. However, there is a paucity of research investigating the disparities in their quality. This study evaluated the primary physiological indicators of both wild and cultivated *O. sinensis*. It also employed proteome and untargeted metabolome approaches to elucidate the differences in quality and underlying mechanisms between the two types. The results revealed that the contents of key representative components, including polysaccharide, crude protein, adenosine, and mannitol, were higher in wild *O. sinensis* than in cultivated *O. sinensis*. A total of 499 differentially expressed proteins (DEPs), including 117 up-regulated and 382 down-regulated DEPs, were identified in wild and cultivated *O. sinensis*. Additionally, 369 up-regulated differentially accumulated metabolites (DAMs) and 737 down-regulated DAMs were also identified. Wild *O. sinensis* had higher relative levels of lysophospholipid metabolites, while cultivated *O. sinensis* had higher relative levels of aldehydes and carboxylic acids. Correlation analysis revealed that different habitats altered 47 pathways shared between the proteome and metabolome, including carbohydrate metabolism and energy metabolism. β-glucosidase and α-galactosidase play essential roles in carbohydrate catabolism and may indirectly influence amino acid synthesis through energy metabolic pathways. The differential expression of polyamine oxidase (PAO) could reflect variations in polyamine metabolism and ammonia production between wild and cultivated *O. sinensis*. These variations may consequently affect nitrogen homeostasis and the biosynthesis of nitrogen-containing compounds, ultimately leading to differences in nutritional quality. In conclusion, these findings offer a novel perspective on the applications of *O. sinensis* and serve as a reference for the targeted development of cultivated *O. sinensis*.

## 1. Introduction

*Ophiocordyceps sinensis* (Berk.) G.H. Sung, J.M. Sung, Hywel-Jones, and Spatafora (Ascomycota, Ophiocordycipitaceae) (syn. *Cordyceps sinensis*) is an exceptional traditional medicinal fungus endemic to the Qinghai–Tibet Plateau, renowned for its remarkable therapeutic properties [1]. Both ancient Chinese medicine and modern literature have highlighted the potential of *O. sinensis* in nourishing the kidney and tonifying the lungs [2], as well as its immunomodulating [3], antidepressant [4], anti-inflammatory [5], intestinal flora-regulating [6], cardiovascular-protecting [7], and anti-apoptotic [8] effects. Due to its medicinal properties, *O. sinensis* has been extensively harvested and consumed as a rejuvenating tonic [9]. Consequently, the excessive exploitation of wild *O. sinensis* resources and the resultant degradation of its ecological habitat have precipitated a substantial decline in its yield, rendering wild *O. sinensis* increasingly scarce [10]. Significant advancements have been made in the cultivated *O. sinensis* to meet the increasing public demand, resulting in its growth and physiological characteristics resembling those observed in its wild counterpart [11]. However, wild and cultivated *O. sinensis* growing in diverse geographical regions may exhibit distinct metabolic profiles due to variations in soil conditions and climate factors [12], thereby influencing the composition and diversity of bioactive constituents with potential medicinal properties [13].

Previous studies have shown that composition determination and morphological identification were the focus of research on wild and cultivated *O. sinensis* [14]. Nevertheless, a comparative analysis of the compositions fails to elucidate the fundamental mechanisms underlying the observed differences. With the advancement of high-throughput sequencing technology [15], the integrated application of omics technology can effectively elucidate disparities in biological characteristics and establish a groundwork for subsequent targeted technologies to be efficiently employed [16]. Proteomics combined with metabolomics, in particular, has been widely used to dissect causal relationships in biological systems, with applications spanning disease research [17] and food science [18]. Previous studies have demonstrated that proteomics and metabolomics techniques can elucidate the changes in the natural air-drying of *Agaricus sinodeliciosus,* and it was revealed that pathways associated with membrane transport, organic acid metabolism, and amino acid metabolism were significantly enriched under drying conditions [19]. Similarly, as a plant that can be used both as medicine and food [20], proteomics and metabolomics analyses phenylalanine/tyrosine/tryptophan biosynthesis and flavonoid metabolic pathways as key drivers of compositional variations across *Scutellaria baicalensis* growth stages, providing a basis for optimizing harvest timing [21]. For ginseng, proteomics studies have shown higher medicinal amino acid levels in wild ginseng versus cultivated species, attributed to increased accumulation of amino acid metabolism-related enzymes and derivatives, including glutamate decarboxylase, S-adenosylmethionine, and methionine synthase [22]. Furthermore, quantitative multi-omics further showed that elevated ginsenosides and phytosterols in wild ginseng stemmed from increased activity of biosynthesis-related enzymes [23]. *O. sinensis* is a medicinal fungus that can be artificially cultivated and has been extensively researched for its active components, efficacy, and mycelium fermentation culture [11]. Comparative morphological and transcriptomic analyses revealed significant differences in both the morphology and unsaturated fatty acid content of *O. sinensis*, with wild *O. sinensis* exhibiting distinct morphological variations across different environments and demonstrating substantially higher levels of unsaturated fatty acids than cultivated *O. sinensis* under low-temperature stress conditions [13]. In addition, previous research has demonstrated that different wild and cultivated *O. sinensis* proteins mainly involve energy production/transformation, amino acid transport/metabolism, and transcriptional regulation [24]. Integrated transcriptomic and metabolomic analyses revealed substantial variations in metabolic profiles between wild and cultivated *O. sinensis*, particularly evident in the differential accumulation of amino acids and their derivatives as well as carbohydrates and their derivatives [25]. Therefore, multi-omics data integration is crucial for elucidating accumulation differences in medicinal active components (e.g., polysaccharides, adenosine, mannitol) and lipid-based nutritional components between wild and cultivated *O. sinensis*, while also facilitating deeper insights into their corresponding biosynthetic pathways.

Primary and secondary metabolites serve as the fundamental constituents of traditional Chinese medicine (TCM) [26], with secondary metabolites being particularly susceptible to environmental factors and adversity, leading to variations in their contents [27]. Previous studies have indicated that the accumulation of metabolites in *O. sinensis* can be profoundly influenced by environmental conditions and growth stages [28]. Enzymes commonly play a crucial role in facilitating biological functions; therefore, the differential expression of enzymes can modulate the levels of active compounds by regulating the synthesis and accumulation of secondary metabolites [29]. Simultaneously, the coordinated regulation of multiple metabolic pathways by multiple enzymes plays a pivotal role in determining the composition of active ingredients [21]. Therefore, the differences and mechanisms in the accumulation of active compounds in wild and cultivated *O. sinensis* can be effectively evaluated through high-throughput proteomic techniques and metabolomics. In this study, proteomics and untargeted metabolomics were used to systematically compare the metabolite profiles and protein profile of wild and cultivated *O. sinensis*, to elucidate the processes affecting the accumulation of bioactive substances and the overall quality of *O. sinensis*.

## 2. Materials and Methods

### 2.1. Material

Fresh samples of wild *O. sinensis* (CS) were procured from Qinghai Baohuitang Biotechnology Co., Ltd. (Xining, China), with collection site in Zaduo County, Yushu City, Qinghai Province, China (33°08′15″ N, 95°38′12″ E), while fresh samples of cultivated *O. sinensis* (ACS) were obtained from Shenzhen Dongyangguang Industrial Development Co., Ltd. (Shenzhen, China). Following the acquisition, all samples were air dried under light-shielded conditions, with the temperature maintained between 22 and 24 °C. No interventions were performed during the drying process. The mass of the samples was measured at 3 h intervals until the difference between two consecutive weighing was less than or equal to 0.01 g, after which they were preserved in an ultra-low-temperature freezer at –80 °C pending subsequent analytical procedures.

### 2.2. Determination of Polysaccharide, Crude Protein, Mannitol, and Adenosine

Polysaccharide content determination kit (JC0410-M) was purchased from Nanjing Integrated Measurement Biotechnology Co., Ltd. After stabilizing at 105 °C, the samples were dried to constant weight at 65 °C, pulverized using a FW100 pulverizer (Tianjin Taisite, Tianjin, China), passed through a 40-mesh sieve, and then weighed. Briefly, 0.05 g of drying sample was added in 1 mL of water and thoroughly homogenized and extracted in a water bath at a temperature of 100 °C for 2 h. After cooling at a centrifugal force of 10,000×*g* and centrifuging for 10 min, an aliquot of 0.2 mL supernatant was absorbed and slowly mixed with 0.8 mL anhydrous ethanol, left overnight at 4 °C, and then centrifuged at 10,000× *g* for 10 min. The supernatant was discarded, and the precipitation was dissolved by adding 1 mL of double distilled water, followed by thorough mixing. Blank, sample, and standard tubes were prepared according to the instructions. They were immersed in water at a temperature of 95 °C for 20 min before naturally cooling down to room temperature and then followed by an assessment of absorbance at 490 nm after mixing. The standard curve was established with glucose as the standard material for detecting the polysaccharide content. The linear relationship is y = 8.8038x − 0.0257, R^2^ = 0.9976. Determination of the sample absorbance was plugged into the equation to get the x value (mg/mL).

Polysaccharide content (mg/g) = x × V_1_/V_2_ × V_3_/W = 5x/W

where V_1_ is the volume of alcohol redissolved after precipitation; V_2_ is the volume for alcohol precipitation; V_3_ is the volume of water added during extraction; and W is the sample mass (g).

Protein content was detected by the national food safety standard (GB_T5009.5-2016) [30]. The crude protein content was determined by sulfuric acid digestion and the Kjeldahl nitrogen determination method [31]. The content of mannitol was determined by High-Performance Liquid Chromatography (HPLC) (Thermo UltiMate 3000, La Jolla, CA, USA), the chromatographic column was HP-Amino (250 mm × 4.6 mm), and the mobile phase was as follows: the flow rate of 70% acetonitrile was 1 mL/min, the column temperature was 40 °C, the temperature of the differential detector was 40 °C, and the sample size was 10 μL. Weigh the sample of about 0.1 g, add 1 mL of extraction solution, homogenize at low temperature, extract by ultrasound for 60 min, and centrifuge to obtain a supernatant. The content of adenosine was determined by HPLC (Rigol L-3000) with Sepax C18 reverse phase column (250 mm × 4.6 mm, 5 μm). The mobile phase was methanol–water = 15:85. The flow rate was 1 mL/min, the column temperature was 30 °C, and the sample size was 10 μL. Three biological replicates were performed for each indicator to ensure result reliability and reproducibility.

### 2.3. Proteomics Profiling

Protein samples were prepared through mechanical homogenization in a lysis solution containing 8 M urea, 1% SDS, and protease inhibitors. Tissue fragmentation was accomplished using three 40 s cycles of mechanical grinding. The homogenate was then incubated at 0–4 °C for 30 min, with brief vortexing at 5 min intervals. After centrifugation (16,000× *g*, 4 °C, 30 min), protein concentration in the supernatant was determined by BCA protein assay kit (Thermo Fisher Scientific, Shanghai, China).

All samples had three independent biological replicates. Protein aliquots (100 μg) were dissolved in 100 mM triethylammonium bicarbonate (TEAB) buffer, followed by reduction using 10 mM Tris (2-carboxyethyl) phosphine at 37 °C for 1 h. Subsequent alkylation was performed with 40 mM iodoacetamide under dark conditions at ambient temperature for 40 min. Protein precipitation was achieved by adding ice-cold acetone (6:1 *v*/*v* acetone-to-sample ratio) and maintaining it at −20 °C for 4 h. After centrifugation at 10,000× *g* for 20 min, the pellet was resuspended in 100 µL of 100 mM TEAB and subjected to enzymatic digestion using trypsin (1:50 *w*/*w* enzyme-to-protein ratio) at 37 °C for 12–16 h. Peptide desalination and quantification were performed according to Jiang et al., with peptide concentration determined using a commercial quantification kit (23275, Thermo Fisher Scientific) [32]. Equal amounts of peptide samples were combined and concentrated using vacuum centrifugation, followed by re-suspension in UPLC loading buffer (Phase A: 2% acetonitrile, pH 10; Phase B: 80% acetonitrile, pH 10). To enhance proteomic depth, the mixed peptides were fractionated using a Vanquish Flex binary UHPLC chromatography system (Thermo, La Jolla, CA, USA) equipped with an Acquity UPLC BEH C18 Column (1.7 µm, 2.1 mm × 150 mm, Waters, Milford, MA, USA). The separation process involved a 47 min gradient elution at a 200 μL/min flow rate. The elution gradient was programmed as follows: 0–16 min, 0–0% B; 16–17 min, 0–3.8% B; 17–34 min, 3.8–24% B; 34–37 min, 24–30% B; 37–38 min, 30–43% B; 38–39 min, 43–100% B; 39–44 min, 100–0% B; and 44–47 min, maintaining 0% B. Six fractions were collected for further analysis from the mixed sample. Based on peptide quantification results, the DIA mass detection can be carried out according to the method of Mu et al [33]. Briefly, The C18 column (75 μm × 25 cm) was equilibrated with solvent A (2% ACN, 0.1% formic acid) and solvent B (80% ACN, 0.1% formic acid). Peptides were eluted with the following gradient: 0–45 min, 3–28% B; 45–50 min, 28–44% B; 50–55 min, 44–90% B; 55–60 min, 90% B, at 250 nL/min. Data were acquired using the timsTOF Pro2 mass spectrometer (Bruker, Billerica, MA, USA) in DIA-PASEF mode. The DIA-PASEF raw data were processed using Spectronaut software (version 14.10.201222.47784, Biognosys AG, Zürich, Switzerland). According to Mu et al., shared and modified peptides were excluded, and peak areas were calculated and summed to generate the final quantitative results [33].

### 2.4. Metabolic Profiling

For the metabolomics analysis, six independent biological replicates were performed for each sample to ensure the reliability and reproducibility of the results. A 50 mg aliquot of each sample was placed into a 2 mL centrifuge tube, and metabolite extraction was performed using 400 μL of an extraction solvent (methanol–water = 4:1, *v*/*v*) supplemented with 0.02 mg/mL L-2-chlorophenylalanine as an internal standard. The samples were homogenized using a Wonbio-96c cryogenic tissue grinder (Shanghai Wanbo Biotechnology Co., Ltd., Shanghai, China) at −10 °C and 50 Hz for 6 min, followed by ultrasonic extraction at 5 °C and 40 kHz for 30 min. After incubation at −20 °C for 30 min, the samples were centrifuged at 13,000× *g* for 15 min (4 °C), and the supernatant was collected for LC-MS/MS analysis. To ensure analytical consistency, equal volumes of all samples were pooled to prepare a quality control (QC) sample, which was used to monitor system stability throughout the analysis.

LC-MS/MS analysis was performed using a SCIEX UPLC-Triple TOF 5600 (SCIEX, Framingham, MA, USA) system with an ACQUITY HSS T3 column (100 mm × 2.1 mm, 1.8 μm) at Majorbio Bio-Pharm Technology Co. Ltd. (Shanghai, China). The mobile phases consisted of 0.1% formic acid in water–acetonitrile (95:5, *v*/*v*) (solvent A) and 0.1% formic acid in acetonitrile–water (47.5:47.5:5, *v*/*v*/*v*) (solvent B). For positive ion mode, the gradient was 0–3 min, 0–20% B; 3–4.5 min, 20–35% B; 4.5–5 min, 35–100% B; 5–6.3 min, 100% B; 6.3–6.4 min, 100–0% B; and 6.4–8 min, 0% B. For negative ion mode, the gradient was 0–1.5 min, 0–5% B; 1.5–2 min, 5–10% B; 2–4.5 min, 10–30% B; 4.5–5 min, 30–100% B; 5–6.3 min, 100% B; 6.3–6.4 min, 100–0% B; and 6.4–8 min, 0% B. The flow rate was 0.4 mL/min, and the column temperature was maintained at 40 °C. The UPLC system was connected to a Triple TOF™ 5600^+^ mass spectrometer (SCIEX, Framingham, MA, USA) with an ESI source operating in both positive and negative modes. Key parameters were optimized as follows: source temperature, 550 °C; curtain gas (CUR), 30 psi; Ion Source Gas1 and Gas2, 50 psi; ion-spray voltage (ISVF), −4000 V (negative mode) and 5000 V (positive mode); declustering potential, 80 V; collision energy (CE), 20–60 eV (rolling for MS/MS). Data were acquired in Information Dependent Acquisition (IDA) mode, with a mass range of 50–1000 *m*/*z*.

### 2.5. Bioinformatics Analysis

Proteomic data analysis was conducted using the Majorbio Cloud Platform (https://cloud.majorbio.com, accessed on 2 November 2024). Differential protein expression between groups was assessed using the R package (Version 4.1.3) Student’s *t*-test (https://www.r-project.org/, accessed on 2 November 2024), with significance thresholds set at |log2 Fold Change| ≥ 1 and *p*-value < 0.05. Functional annotation of proteins was performed using Gene Ontology (GO, http://geneontology.org/, accessed on 2 November 2024) and the Kyoto Encyclopedia of Genes and Genomes pathway database (KEGG, http://www.genome.jp/kegg/, accessed on 2 November 2024). Differentially expressed proteins (DEPs) were further analyzed for GO and KEGG enrichment. Protein–protein interaction networks were generated using STRING (v11.5) with a minimum interaction confidence score of 0.7.

The UHPLC-MS raw data were processed using Progenesis QI software version 3.0 (Waters, Milford, MA, USA) for baseline filtering, peak identification, integration, retention time correction, and alignment, generating a data matrix with sample names, *m*/*z*, retention time, and peak intensities. Metabolites were identified by querying the Human Metabolome Database (HMDB, http://www.hmdb.ca/, accessed on 2 November 2024), Metlin (https://metlin.scripps.edu/, accessed on 2 November 2024), and the MJDB database (Majorbio Biotechnology Co., Ltd., Shanghai, China). Using the R package “ropls” (v1.6.2), principal component analysis (PCA) and orthogonal partial least squares-discriminant analysis (OPLS-DA) were performed, and permutation testing (200 permutations) was performed to evaluate model overfitting. Differentially accumulated metabolites (DAMs) were identified based on variable importance in the projection (VIP) >1, |log2FC| ≥ 1, and *p* < 0.05 from the OPLS-DA model and Student’s *t*-test. DAMs were mapped to biochemical pathways using KEGG (Version 85.0) (http://www.genome.jp/kegg/, accessed on 2 November 2024) for metabolic enrichment and functional classification.

Integrated proteome–metabolome analysis was performed on the Majorbio Cloud Platform (https://cloud.majorbio.com, accessed on 2 November 2024) to identify shared KEGG pathways between DEPs and DAMs. Fisher’s exact test-based enrichment analysis was applied to DEPs and DAMs to pinpoint biologically relevant processes. A correlation network was constructed using Pearson correlation coefficients, with nodes retained if at least one group of correlations met |corr| > 0.95 and *p* < 0.05. For visualization, the top 30 nodes with the highest absolute log2FC values were selected from each omics dataset, excluding proteomic nodes with extreme FC values. After confirming that all datasets met the normality assumption via the Shapiro–Wilk test, Student’s *t* test (SPSS 26.0) was conducted, and results were visualized using GraphPad Prism (v10.0).

## 3. Results

### 3.1. Analysis of Key Active Components of Wild and Cultivated O. sinensis

**Figure 1:**
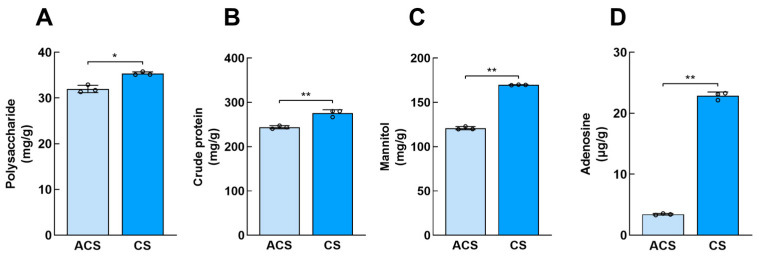
Comparison of active components of wild and cultivated *O. sinensis*. (**A**) Polysaccharide content; (**B**) crude protein content; (**C**) mannitol content; (**D**) adenosine content. “*” indicates a significant difference (*p* < 0.05); “**” indicates an extremely significant difference (*p* < 0.01).

The quality evaluation of wild and cultivated *O. sinensis* was conducted by determining and analyzing their primary bioactive constituents. It was found that the polysaccharide content of CS was 35.35 ± 0.38 mg/g and that of ACS was 31.99 ± 0.81 mg/g (Figure 1A). The crude protein content of CS was 275.74 ± 7.71 mg/g, and the crude protein content of ACS was 243.93 ± 3.47 mg/g, which is 1.13 times that of ACS (Figure 1B). In addition, the mannitol content of CS was 169.71 ± 0.29 mg/g, which is 1.4 times that of ACS (Figure 1C). Therefore, there were differences in polysaccharide content (*p* < 0.05), but there were significant differences in crude protein, mannitol, and adenosine (*p* < 0.01). As a quality control index of Chinese Pharmacopeia [34], we found that the content of adenosine in CS was as high as 22.86 ± 0.62 μg/g, while that in ACS was only 3.43 ± 0.13 μg/g, indicating that different habitats may have different degrees of influence on the synthesis of adenosine (Figure 1D). We hypothesized that the disparities in polysaccharide, crude protein, and mannitol contents between CS and ACS could be attributed to variations in the enzymes involved in synthesizing related metabolites and the subsequent accumulation of these metabolites. Consequently, we conducted further analyses on their proteome and metabolome.

### 3.2. Proteomic Analyses of Wild and Cultivated O. sinensis

**Figure 2:**
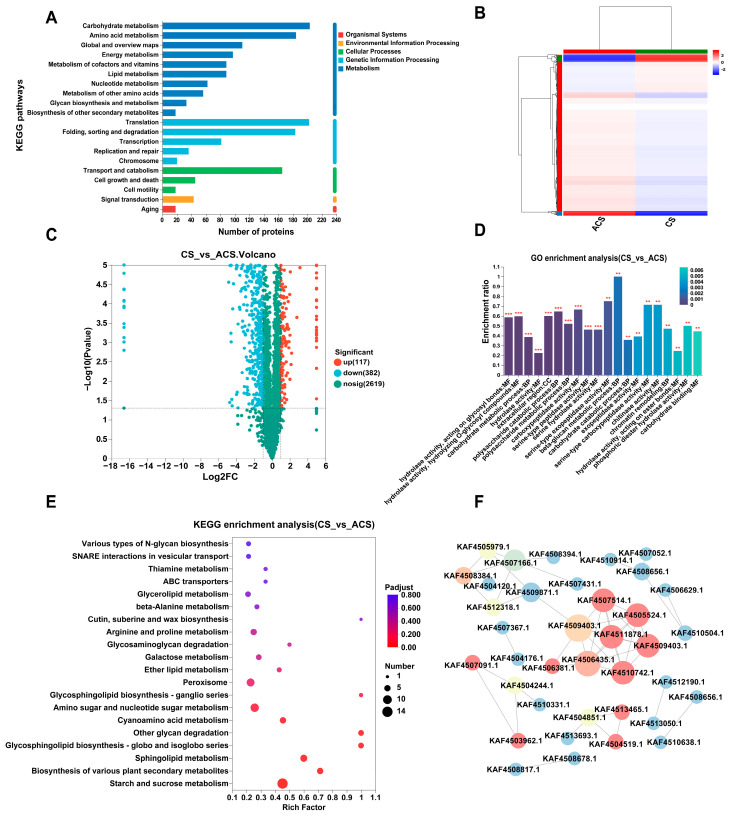
Proteomic profiling of wild and cultivated *O. sinensis.* (**A**) KEGG pathway functional classification and annotation. (**B**) Cluster analysis of differential proteins, where each column in the figure corresponds to a sample, and each row represents a protein. The color gradient in the figure reflects the relative expression levels of proteins within their respective sample groups. (**C**) Volcano plots present the differences between the two groups, where the *x*-axis indicates the log2-fold change in protein expression between the two samples, while the *y*-axis represents the −log10 (*p*-value) derived from the statistical test of protein expression differences. Both axes have been subjected to logarithmic transformation for better visualization. Each point in the plot corresponds to a specific protein, with leftward points indicating down-regulated proteins and rightward points indicating up-regulated proteins. (**D**) GO functional enrichment analysis, where the horizontal axis represents the GO term, the vertical axis represents the enrichment rate, and the color gradient of the columns indicates the significance of enrichment. Those with *P* or FDR < 0.001 are marked with “***” and those with *P* or FDR < 0.01 are marked with “**”. (**E**) KEGG pathway analysis of DEPs, where the *x*-axis denotes the enrichment rate, while the *y*-axis indicates KEGG pathways. Each bubble in the figure corresponds to a specific KEGG pathway, and the size of the bubble reflects the number of enriched proteins in the protein cluster associated with that pathway. (**F**) PPI network analyses, where red indicates the stronger interaction relationship between nodes, blue represents the weaker one, while yellow and green denote the moderate interaction relationship.

Approximately 26,081 unique peptides corresponding to 3118 proteins were successfully identified through LC-MS/MS mass spectrometry identification. The PCA results demonstrated that the wild and cultivated *O. sinensis* exhibited distinct clustering within their respective categories and were significantly separated from each other. The first principal component (PC 1) explained 60.40% of the variance, while the second principal component (PC 2) accounted for 17.80% of the variance (Figure S1). The identified proteins were annotated using the KEGG database, revealing that in metabolism, there was a predominant enrichment of proteins involved in carbohydrate metabolism and amino acid metabolism. Within genetic information processing, the functions of proteins primarily encompass translation, folding, sorting, and degradation (Figure 2A). The quantitative expression analysis and screening threshold led to the identification of 499 DEPs (Table S1), with 117 showing up-regulation and 382 displaying down-regulation. We performed clustering analysis on the expression profiles of the selected DEPs and found that there are significant differences in the protein expression profiles between CS and ACS (Figure 2B). Meanwhile, the DEPs were visualized using a volcano plot (Figure 2C). The GO function was subjected to a comprehensive enrichment analysis based on the database. According to their functional characteristics, 499 DEPs were classified into biological process (BP), cellular component (CC), and molecular function (MF) categories (Figure 2D). The main functional categories in BP classification were the carbohydrate metabolic process, β-glucan metabolic process, and polysaccharide catabolic process. For MF, the most abundant groups are hydrolase activity (acting on glycosyl bonds), hydrolase activity, and carboxypeptidase activity; in CC, the extracellular region has the highest content.

KEGG pathway enrichment analysis was employed to elucidate the molecular pathways encompassing DEPs. A total of 499 proteins were assigned to 78 pathways, with the majority being distributed in starch and sucrose metabolism, sphingolipid metabolism, glycosphingolipid biosynthesis, and glycan degradation (Figure 2E). The findings suggested that the quality of CS and ACS was associated with alterations in metabolic pathways involving glucose and lipid metabolism enzymes. Furthermore, we selected top 50 highly correlated proteins and constructed a protein–protein interaction (PPI) network to elucidate the interactions among key proteins (Figure 2F). After excluding isolated nodes, we identified that pivotal DEPs, including 60S ribosomal protein L31 (KAF4507514.1), ribosomal protein L22e (KAF4511878.1), 60S ribosomal protein L11 (KAF4510742.1), 40S ribosomal protein S21 (KAF4505524.1), KOW domain-containing protein (KAF4509403.1), and RRM domain-containing protein (KAF4508384.1), which were significantly interconnected in the PPI network. These proteins collectively form a ribosome assembly and translation regulation network. Changes in the expression levels of ribosomal proteins can affect translation efficiency, and ribosome activity is closely linked to the synthesis of secondary metabolites. Therefore, differences in the translation processes responding to external signals may represent one of the important factors contributing to the quality disparities between wild and cultivated *O. sinensis*.

### 3.3. Comparative Analyses of the Metabolite Profiles of Wild and Cultivated O. sinensis

**Figure 3:**
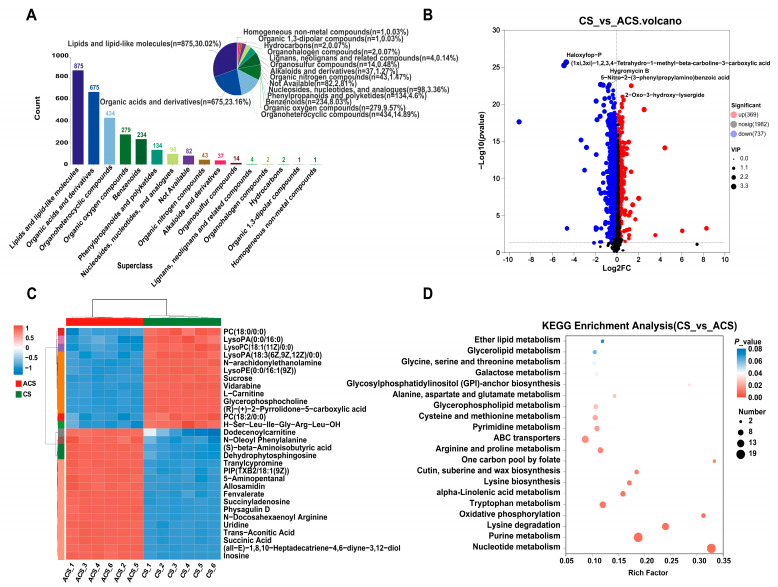
Overview of metabolomics results. (**A**) HMDB classification of all metabolites. (**B**) Volcano plots present the differences between the two groups, where the *x*-axis indicates the fold change in metabolite expression between the two groups, while the *y*-axis denotes the statistical significance of the metabolite expression changes. Each point in the plot corresponds to a specific metabolite, with the size of the point reflecting its VIP value. Red points represent metabolites with significantly up-regulated expression, blue points indicate significantly down-regulated metabolites, and black points represent metabolites with no significant expression differences. (**C**) Clustering heat map of the Top 30 DAMs; each column in the figure corresponds to a specific sample, and each row is associated with a particular metabolite. The color gradient in the figure reflects the relative expression levels of metabolites within the corresponding sample group. (**D**) KEGG pathway analysis of DAMs; the *x*-axis denotes the enrichment rate, and the *y*-axis indicates the KEGG pathways. The size of the bubbles reflects the number of compounds enriched in the corresponding metabolic pathway, whereas the color gradient of the bubbles represents the significance level of the enrichment *p*-values.

Total ion chromatograms (TICs) of the quality control (QC) samples obtained through positive electrospray ionization (ESI^+^) and negative electrospray ionization (ESI^−^) are presented in Figure S2. The results demonstrate a significant overlap between the TIC peaks’ response intensity and retention time, indicating minimal experimental equipment-induced error. In summary, 3088 metabolites were detected from CS and ACS, and according to the HMDB database (Figure 3A), all detected metabolites were divided into 16 superclasses. Among all metabolites, the most abundant superclass was lipids and lipid-like molecules, including 875 metabolites (30.02%), followed by organic acids and derivatives (675 metabolites (23.16%)), and organoheterocyclic compounds (434 metabolites (14.89%)). We conducted a multivariate statistical analysis to identify statistically significant differences in metabolite abundance between CS and ACS. PCA dimensionality reduction and OPLS-DA analysis demonstrated a distinct separation between the two groups (Figure S3). The first principal component accounted for 50.60% of the total variability, while the second principal component explained 39.20% of the dataset’s overall variability, indicating complete segregation of samples based on these components and highlighting significant disparities between CS and ACS. To validate model reliability and exclude overfitting, a permutation test with 200 random permutations was performed. The results showed that the intercept of the permutation plot for Q^2^ was <0.05, and R^2^Y remained consistently high (Figure S3).

After normalizing the metabolomic results, comparable DAMs were identified in CS and ACS using volcano plots (Figure 3B), which were determined based on the cutoff criteria of VIP > 1, |log2FC| ≥ 1, and *p*-value < 0.05. The analysis revealed an up-regulation of 369 DAMs and a down-regulation of 737 DAMs (Table S2). Our cluster analysis of the Top 30 DAMs found that CS was rich in lipids, such as LysoPA (0:0/16:0) and LysoPC (18:1 (11Z)/0:0), and the content of 5-Aminopentanal and succinic acid in ACS was significantly up-regulated (Figure 3C). KEGG database was employed to analyze pathway enrichment of key metabolic processes and investigate the enrichment of essential metabolic pathways further. The results demonstrate that these DAMs are significantly enriched in 86 pathways, primarily associated with nucleotide metabolism, purine metabolism, lysine degradation, and tryptophan metabolism (Figure 3D).

### 3.4. Combined Proteomic and Metabolomic Analyses

**Figure 4:**
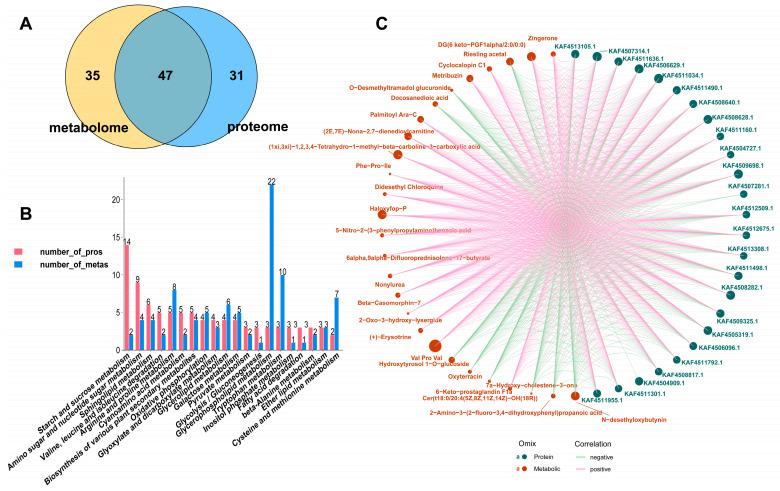
Joint analysis of metabolome and proteome of DEPs and DAMs. (**A**) Venn diagram of common KEGG pathway for DAMs and DEPs. (**B**) Top 20 common KEGG pathways of DAMs and DEPs enrichment. (**C**) Correlation network analysis was conducted on the top 30 absolute value log2FC of proteome and metabolome; those exhibiting the maximum and minimum FC values were excluded to prevent potential distortion of the visualization results caused by extreme values. Each node denotes either a protein or a metabolite, with red circles representing metabolites and green circles representing proteins. Each line signifies the interaction between proteins and metabolites, where red lines denote positive correlations and green lines denote negative correlations and the width of the lines reflects the *p*-value.

KEGG functional annotation of all identified DEPs and DAMs was conducted to enhance our understanding of the correlation between the metabolomics and proteomics of CS and ACS. Venn diagram depicted a total of 47 KEGG pathways in the proteomic and metabolomic data, predominantly encompassing carbohydrate metabolism, lipid metabolism, and amino acid metabolism (Figure 4A). This observation may be closely associated with the variations in synthetic adenosine content between CS and ACS. After conducting a comprehensive analysis of 47 common pathways, we have successfully identified the top 20 KEGG pathways that exhibit the highest abundance of proteins and metabolites (Figure 4B). Integrated omics analysis has revealed that these metabolites and proteins primarily participate in primary and secondary metabolism, including carbohydrate metabolism, lipid metabolism, amino acid metabolism, metabolism of other amino acids, energy metabolism, as well as biosynthesis of other secondary metabolites. Additionally, we have observed that the disparities in the quality of *O. sinensis* cultivated in diverse habitats can primarily be attributed to the strong correlation between its carbohydrate and lipid metabolism and synthesis.

Calculating the Pearson correlation coefficient assesses the degree of association between proteins and metabolites in a sample, enabling a deeper comprehension of the interconnections between metabolomics and proteomics (Figure 4C). The expression level of α-galactosidase (KAF4506629.1) was positively correlated with 2-Oxo-3-hydroxy-lysergide, β-casomorphin-7, nonylurea, Phe-Pro-Ile, (2E,7E)-nona-2,7-dienedioylcarnitine, and palmitoyl Ara-C. In contrast, it showed a negative correlation with docosanedioic acid and O-desmethyltramadol glucuronide. Furthermore, β-glucosidase (KAF4511498.1) exhibits a positive correlation with β-casomorphin-7, nonylurea, haloxyfop-P, didesethyl chloroquine, and Phe-Pro-Ile. In addition, analogous to α-galactosidase, it demonstrates a negative correlation with docosanedioic acid and O-desmethyltramadol glucuronide. Furthermore, Val-Pro-Val and Phe-Pro-Ile, along with proteins including GH16 domain-containing protein (KAF4505319.1), six-hairpin glycosidase (KAF4504727.1), FAD-binding PCMH-type domain-containing protein (KAF4506096.1), α-galactosidase (KAF4506629.1), M6 family metalloprotease domain-containing protein (KAF4507314.1), lysM domain-containing protein (KAF4508282.1), carboxylic ester hydrolase (KAF4508628.1), and Duf1680 domain-containing protein (KAF4508640.1), exhibit a positive correlation. It is suggested that short peptides may be involved in the distribution of sugar metabolism products, and affect the balance between polysaccharide synthesis and secondary metabolism. Furthermore, our research demonstrates that several lipids, including docosanedioic acid, palmitoyl Ara-C, and DG (6 keto-PGF1α/2:0/0:0), exhibit a positive correlation with carboxylic ester hydrolase and extracellular phospholipase C. This result indicates that the synergistic activation of multiple proteins may enhance the activity of hydrolases and promote the expression of genes related to secondary metabolism, ultimately leading to the accumulation of metabolites. In conclusion, disparities in key enzymes of carbohydrate and lipid metabolism between wild and cultivated *O. sinensis* may regulate the metabolic regulatory network, ultimately resulting in variations in the accumulation of bioactive substances.

### 3.5. Specifically Expressed Metabolic Process-Related Proteins and Metabolites in Wild and Cultivated O. sinensis

**Figure 5:**
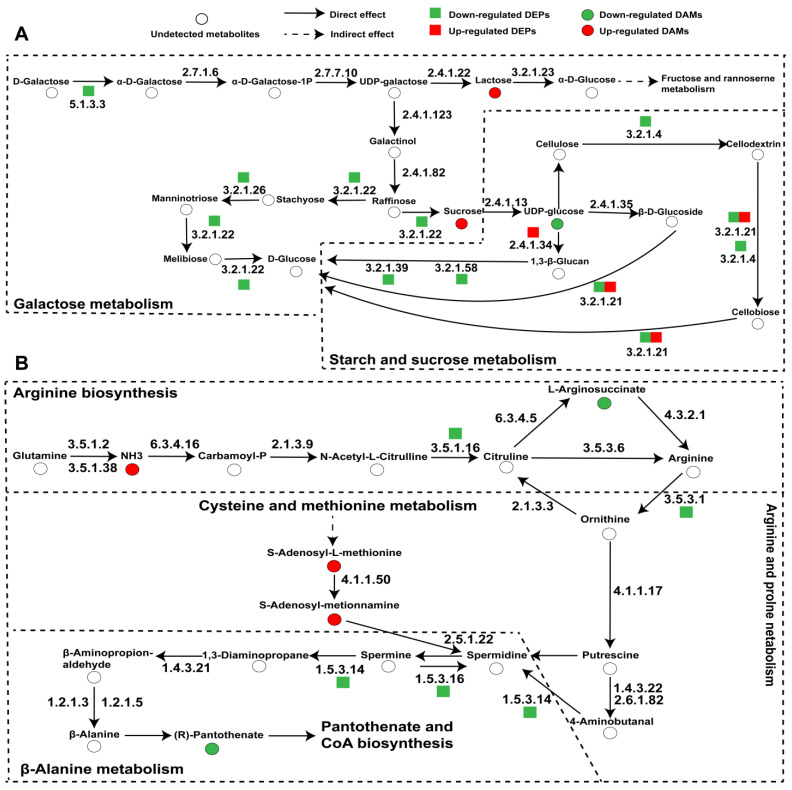
Schematic diagram of critical pathways of wild and cultivated *O. sinensis*. (**A**) Starch and sucrose metabolism; galactose metabolism; (**B**) arginine biosynthesis; cysteine and methionine metabolism; arginine and proline metabolism; β-alanine metabolism; pantothenate and CoA biosynthesis. Red dots represent the up-regulated DAMs, green dots represent the down-regulated DAMs, red squares represent the up-regulated DEPs, green squares represent the down-regulated DEPs, and white dots represent the metabolites or proteins with no significant difference.

Elucidation of the synthetic pathways of key components is crucial for a more comprehensive investigation into the quality disparities between CS and ACS. Through our comparative metabolomic and proteomic analyses, we characterized the differential regulation of key metabolic pathways involved in starch and sucrose metabolism, amino sugar and nucleotide sugar metabolism, sphingolipid metabolism, valine, leucine, and isoleucine degradation, as well as arginine and proline metabolism. The findings contribute to a better understanding of the intricate regulatory mechanisms governing the accumulation and synthesis of components in both CS and ACS. Many proteins have been noted to be involved in starch and sucrose metabolism (Figure 5A). In starch and sucrose metabolism, 1,3-β-glucan synthase [EC 2.4.1.34] and β-glucosidase [EC 3.2.1.21] are significantly up-regulated. In galactose metabolism, α-galactosidase [EC 3.2.1.22] and β-fructofuranosidase [EC 3.2.1.26] were significantly decreased. These findings indicate that the accumulation of sucrose and starch in both CS and ACS across various habitats may be influenced by external factors, resulting in variations in their growth and supply capacity. In addition, we speculate that the main differences between CS and ACS may be due to changes in the metabolic pathways of amino acids (Figure 5B). These include arginine biosynthesis, cysteine and methionine metabolism, arginine and proline metabolism, and other pathways. Enzymes related to arginine synthesis and metabolism are regulated. In the arginine biosynthesis, acetylornithine deacetylase [EC 3.5.1.16] and arginase [EC 3.5.3.1] are significantly down-regulated. Polyamine oxidase (PAO) [EC 1.5.3.14/1.5.3.16] was down-regulated in arginine and proline metabolism, suggesting differences in ammonia metabolism and nitrogen balance maintenance between CS and ACS.

## 4. Discussion

The medicinal value of *O. sinensis* has gained widespread recognition among common people, prompting increased attention to quality differences between CS and ACS. The impact of environmental stress on the species and composition of secondary metabolites in *O. sinensis* was well recognized. Therefore, we initially performed absolute quantification of four crucial constituents in *O. sinensis*. The absolute quantitative content of polysaccharide, crude protein, mannitol, and adenosine in CS was higher than in ACS. Previous research has demonstrated that varying altitudes significantly influence the metabolic profile of CS, potentially resulting in divergent medicinal effects [35]. However, *O. sinensis* growing in different habitats may exhibit more pronounced variations. Numerous studies have demonstrated that plants or fungi inhabiting high altitudes encounter the challenges of temperature fluctuations and ultraviolet radiation [36], significantly impacting their nutrient acquisition and secondary metabolite synthesis [37]. CS primarily inhabits high-altitude habitats and undergoes more pronounced environmental fluctuations that potentially influence the accumulation of specific primary metabolites such as polysaccharides and crude proteins. Previous studies also revealed variations in the accumulation and synthesis of flavonoid metabolites in *Lycium barbarum* grown at different altitudes, which can be primarily attributed to differential expression of chalcone isomerase (*CHI*), chalcone synthase (*CHS*), and flavonol synthase (*FLS*) genes that play crucial roles in regulating flavonoid biosynthesis [27]. Additionally, Pan et al. investigated altitude gradients and discovered that high altitudes stimulated nitrogen metabolism in *Asarum* leaves and carbon metabolism in roots, influencing the metabolic pathways of phenolic substances such as syringic acid, vanillic acid, and ferulic acid to enhance organic acid metabolism [38]. Our results demonstrate a significant positive correlation between the polysaccharide contents in CS and ACS and the expression levels of β-glucosidase [EC: 3.2.1.21] and callose synthase [EC: 2.4.1.34]. β-glucosidase is essential for hydrolyzing cellulose into glucose, which serves as a critical carbon source supporting cellular metabolism [39]. Through sugar metabolic pathways, this glucose is metabolized into nucleotide-sugars that serve as activated precursors for polysaccharide synthesis. Moreover, callose synthase regulates the chain length and branching patterns of polysaccharides, thereby promoting the biosynthesis of β-1,3-glucan [40]. Therefore, we have observed that the disparities in the content of polysaccharide, crude protein, mannitol, and adenosine quantities between CS and ACS may potentially be attributed to the genes or enzymes governing product survival.

Protein translation and post-translational modifications enable accurate analysis of fungal protein expression across diverse environments, including distinct growth conditions and tissue morphologies [41]. Previous studies have demonstrated that low-temperature stress in edible fungi significantly impact the production of proteins associated with carbohydrate metabolism and energy metabolism within the fruiting bodies of *Volvariella volvacea*, thereby disrupting energy metabolism, membrane lipid metabolism, and reactive oxygen metabolism [42]. Our findings indicated that the disparity between CS and ACS primarily resides in alterations of carbohydrate metabolism and amino acid synthesis and metabolism, consistent with the results obtained by predecessors [25]. Cultivating *O. sinensis* in low-altitude areas using ecological technology is well established. However, the harsh high-altitude environment induces significant differences in the metabolic and protein profiles of CS and ACS. Metabolic processes such as starch and sucrose metabolism, glycosphingolipid biosynthesis, and glycan degradation may undergo alterations in response to elevation changes. Enzymes like glucan 1,3-β-glucosidase [EC:3.2.1.39], β-glucosidase [EC:3.2.1.21], and endoglucanase [EC:3.2.1.4] play a critical role in the hydrolysis of polysaccharides to produce D-Glucose. In conjunction with prior studies, it has been demonstrated that the relative concentrations of mannitol, D-arabitol, D-xylitol, and other metabolites in ACS are significantly higher compared to those in CS. The present study also revealed an up-regulation in the expression of enzymes associated with synthetic carbohydrates, while *O. sinensis* exhibited a relatively limited accumulation of carbohydrates. This enhanced energy and substrate acquisition in ACS can be attributed to favorable climatic conditions and optimal nutritional environments in low-altitude cultivation [43]. At high altitudes, fungi and plants modify cell wall components to acclimate to low temperatures, potentially altering the composition and abundance of cell wall polysaccharides [44]. Absolute quantification of polysaccharide content in ACS and CS showed significantly higher levels in the wild variety, potentially due to differences in intracellular and extracellular polysaccharide synthesis capabilities induced by stress adaptation.

The development and utilization of TCM primarily rely on the presence of primary and secondary metabolites [45], which are also indicative of the adaptive responses exhibited by certain medicinal organisms toward environmental tolerance [46]. Metabolomics facilitates the comprehensive and systematic identification of TCM metabolites, thereby establishing a robust theoretical foundation for biomarker discovery [47]. Overall, 3088 metabolites were detected in CS and ACS. According to functional similarity, these metabolites were further divided into 16 superclasses, mainly consisting of lipids and lipid-like molecules, organic acids, and derivatives. While sharing common metabolic profiles, the two groups showed differences in specific bioactive compounds. Numerous substances recognized as medicinal compounds possessing valuable pharmacological properties, such as ceramide, are comparatively higher in CS. As a pivotal bioactive substance, it serves as a secondary messenger molecule in sphingolipid metabolism within the nervous system and plays a crucial role in activating various stress-related enzymes [48]. Additionally, it facilitates cellular apoptosis, regulates cellular immunity, attenuates aging processes, and exhibits anti-tumor properties [49]. Furthermore, the content of L-Acetylcarnitine is relatively high in ACS, exhibiting potential for treating neuropathic pain and exerting antidepressant effects [50]. By employing transcriptome and metabolomics analyses, Zhang et al. revealed that the levels of amino acids and their derivatives, carbohydrates and their derivatives, and phenolic acids in *O. sinensis* were significantly elevated compared to those in wild *O. sinensis* [25]. Conversely, the majority of nucleosides and nucleotides exhibited markedly higher concentrations in CS than in ACS, and the nucleoside differences may be related to *IMPDH*, *AK*, *ADSS*, *guaA*, and *guk*, which affect the synthesis of nucleotides and nucleosides [25]. Interestingly, our quantitative analysis revealed that the adenosine content of CS surpassed that of its cultivated counterpart. As principal metabolites, nucleosides exert pharmacological effects and function as essential energy components and enzyme cofactors in metabolic pathways, playing a pivotal role in fundamental biological processes, such as the synthesis of DNA and RNA [51]. In our study, differential expression analyses revealed a significant enrichment of DAMs in nucleotide and purine metabolism pathways. This is likely attributed to the indispensability of purines, which serve as fundamental constituents of DNA and RNA, energy sources, and cofactors in various biological processes, encompassing energy metabolism, signal transduction, and enzyme activity [52]. Under conditions of low oxygen tension, there is an augmented breakdown of purines leading to the up-regulation of nucleosides and their bases [53]. In summary, the findings mentioned above not only elucidate the disparities in the bioactive constituents of CS and ACS but also furnish crucial evidence for the advancement and innovation of *O. sinensis* resources.

## 5. Conclusions

In this study, metabolic and protein profiles of wild *O. sinensis* and cultivated *O. sinensis* were systematically analyzed and compared, using a combination of proteomics and metabolomics. The results demonstrated higher levels of adenosine, polysaccharide, crude protein, and mannitol in wild *O. sinensis* compared to its cultivated counterpart. The enhanced accumulation of these bioactive compounds in wild *O. sinensis* may underlie its medicinal value and quality. Metabolites and proteins with varying abundance in key pathways such as starch and sucrose metabolism and nucleotide metabolism could potentially serve as targets regulated by natural environmental factors or cultivated domestication. Further analysis of the regulatory relationship between ecological factors and cultivated intervention and their corresponding target genes will establish a robust foundation for optimizing *O. sinensis* germplasm resources and constructing an advanced cultivation technology system for *O. sinensis*.

## Data Availability

The mass spectrometry proteomics data for *Ophiocordyceps sinensis* have been deposited to the ProteomeXchange Consortium (https://proteomecentral.proteomexchange.org, accessed on 4 February 2025) via the iProX partner repository with the dataset identifier PXD060491. Metabolome data for *O. sinensis* has been deposited at MetaboLights (https://www.ebi.ac.uk/metabolights/, accessed on 4 February 2025) with accession no. MTBLS12199.

## Supplementary Materials

The following supporting information can be downloaded at https://www.mdpi.com/article/10.3390/jof11070469/s1, Figure S1: Principal component analysis of proteomics; Figure S2: Total ion current chromatogram; Figure S3: Multivariate statistical analysis diagram; Table S1: Differentially expressed proteins of wild and cultivated *O. sinensis*; Table S2: Differentially accumulated metabolites of wild and cultivated *O. sinensis*.
