# Supplementary figures and images for: Integrated Proteomics and Metabolomics Reveal Regulatory Pathways Underlying Quality Differences Between Wild and Cultivated Ophiocordyceps sinensis
Source: J Fungi (Basel). 2025 Jun 20;11(7):469. doi: 10.3390/jof11070469 (PMC12295709; doi:10.3390/jof11070469)

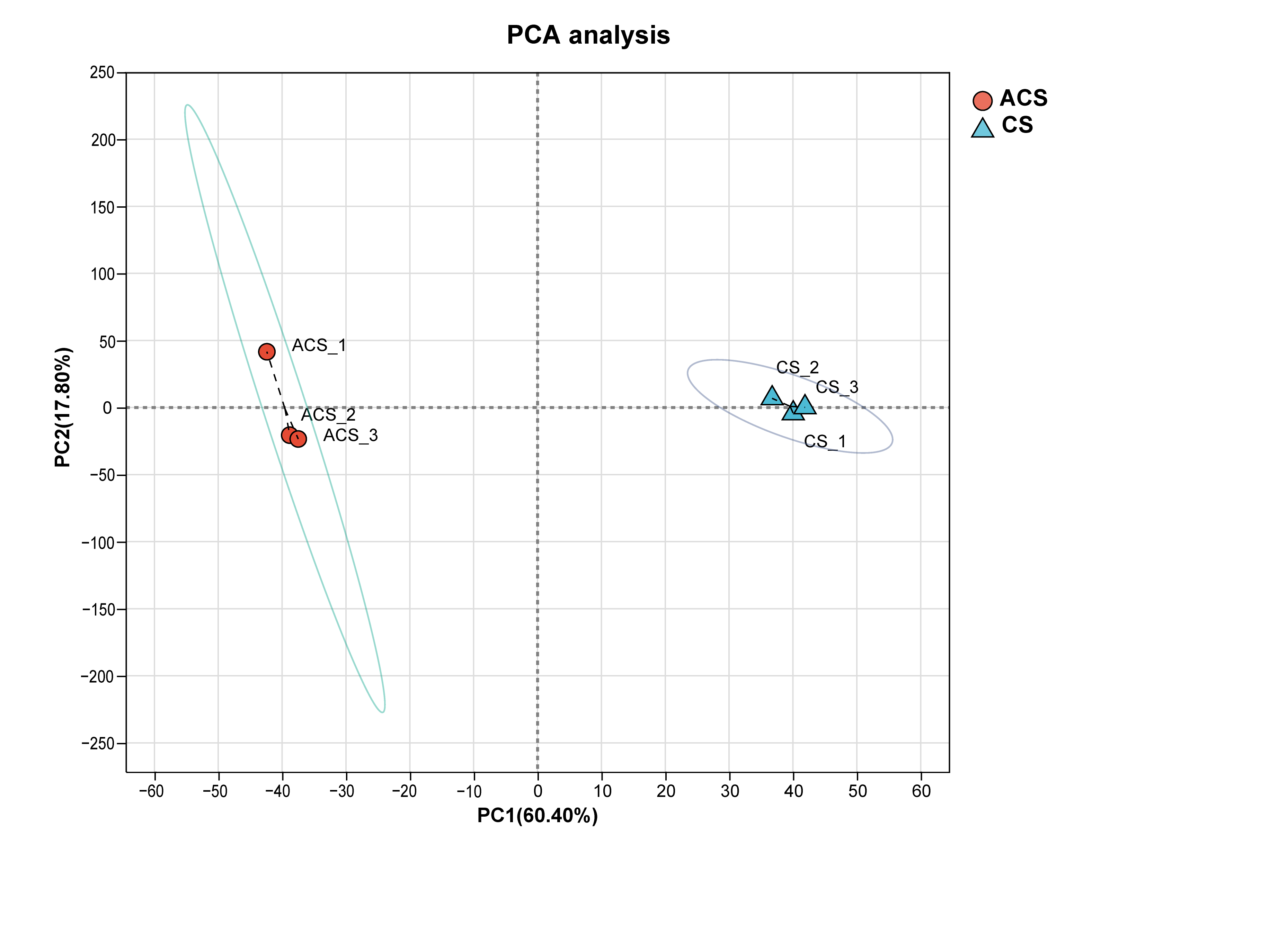

Supplement: Supplementary file 1 [file jof-11-00469-s001.zip › Figure S1.tif]
